# Smart-Contract Aware Ethereum and Client-Fog-Cloud Healthcare System

**DOI:** 10.3390/s21124093

**Published:** 2021-06-14

**Authors:** Abdullah Lakhan, Mazin Abed Mohammed, Ahmed N. Rashid, Seifedine Kadry, Thammarat Panityakul, Karrar Hameed Abdulkareem, Orawit Thinnukool

**Affiliations:** 1 College of Computer Science and Artificial Intelligence, Wenzhou University, Wenzhou 325035, China; Abdullahrazalakhan@gmail.com; 2 College of Computer Science and Information Technology, University of Anbar, Ramadi 31001, Iraq; mazinalshujeary@uoanbar.edu.iq (M.A.M.); rashidisgr@uoanbar.edu.iq (A.N.R.); 3 Faculty of Applied Computing and Technology, Noroff University College, 4608 Kristiansand, Norway; seifedine.kadry@noroff.no; 4 Division of Computational Science, Faculty of Science, Prince of Songkla University, Hat Yai, Songkhla 90110, Thailand; thammarat.p@psu.ac.th; 5 College of Agriculture, Al-Muthanna University, Samawah 66001, Iraq; khak9784@mu.edu.iq; 6 Research Group of Embedded Systems and Mobile Application in Health Science, College of Arts, Media and Technology, Chiang Mai University, Chiang Mai 50200, Thailand

**Keywords:** ethereum, security, privacy, smart-contract, rules, distributed

## Abstract

The Internet of Medical Things (IoMT) is increasingly being used for healthcare purposes. IoMT enables many sensors to collect patient data from various locations and send it to a distributed hospital for further study. IoMT provides patients with a variety of paid programmes to help them keep track of their health problems. However, the current system services are expensive, and offloaded data in the healthcare network are insecure. The research develops a new, cost-effective and stable IoMT framework based on a blockchain-enabled fog cloud. The study aims to reduce the cost of healthcare application services as they are processing in the system. The study devises an IoMT system based on different algorithm techniques, such as Blockchain-Enable Smart-Contract Cost-Efficient Scheduling Algorithm Framework (BECSAF) schemes. Smart-Contract Blockchain schemes ensure data consistency and validation with symmetric cryptography. However, due to the different workflow tasks scheduled on other nodes, the heterogeneous, earliest finish, time-based scheduling deals with execution under their deadlines. Simulation results show that the proposed algorithm schemes outperform all existing baseline approaches in terms of the implementation of applications.

## 1. Introduction

The World Health Organization has now declared the Coronavirus pandemic a global health emergency (WHO). The vast volume of data collected to fight the COVID-19 pandemic poses many security and privacy issues during this period. The integrity of their authentication is essential to guarantee the protection of patient information in the transition process. In innovative healthcare, proper medical data protection is, therefore, becoming equally crucial. They are motivated by these new concepts, methods, theories, and practices focusing on data protection and privacy solutions for intelligent healthcare industries.

The Internet of Medical Things (IoMT) system is an emerging healthcare monitor paradigm that consists of devices, sensors, wireless network, and fog-cloud computing [1,2]. The sensors could be mobile devices, heartbeat sensors, blood pressure IoT sensors, ECG sensors, and many sensors connected with mobile devices [3]. The invention of the 5G communication technology encourages more and more devices to intercommunicate with external resources. Fog-cloud is a cooperative computing network where remote cloud offers via internet and fog computing provide services at the edge of the network [4]. Many healthcare applications have been developed based on the IoMT system, where many patients practised these applications with mobile devices [5]. Simultaneously, healthcare sensors are directly connected to the fog-cloud servers to perform any healthcare task for patients [6]. However, resource-constrained devices offload compute-intensive tasks to the fog cloud for further execution. Generally, devices act as the thin client, and fog-cloud nodes are thick to process all requests with enriching resources in the IoMT system. This offloading mechanism poses many research challenges for healthcare applications, such as data security, malware attack, denial of services and storage issue with unknown external servers [7].

The fog-cloud computing nodes offer distributed paid healthcare services to execute all classes and type of application [7]. There are three cost models for services: on-demand, on-reserved, and spot-instances based on hourly, weekly, monthly, and yearly duration with different prices. Generally, these applications are workflow applications and require a sequence of processes to run users requests into other orders [8]. When needed, they needed services based on their execution, preferably provisionally for an hour or weekly for actions. Therefore, it is a challenge to design a cost-efficient system for healthcare IoMT applications. Generally, vendors offer these paid services (e.g., fog-cloud, such as Amazon, Cloud, Alibaba, and Azure) to run the applications under their Quality of Service (QoS) requirements [9]. Recently, serverless computing is a model for fog-cloud computing execution in which the server runs applications with a function inside containers. The pricing is based on the application’s actual number of resources per number of performances, not on the volume units’ provisioning servers [9].

Data will encrypt in the IoMT application due to security concerns before they are sent to the server. At the same time, the client will face some problems [10]. The main reason for these is that the service provider has to perform data computation to respond to the client’s requests, so the client must provide the server with the key to decrypt the data before performing the appropriate calculation, affecting the cloud’s data confidentiality [10]. Ethereum is a decentralized, open-source blockchain that allows users to create smart contracts with their own rules and regulations. The platform’s native cryptocurrency is Ether. The Ethereum blockchain is the most widely used in the peer-to-peer network and applications for the distributed system. Ethereume blockchain is a group of blocks (data blocks) that are unalterable and well structured, and it records the logs of all transactions, in the form of a file system, the data blocks in the blockchain stored data in each node [11]. Each block contains data for many transactions, the number of which can vary between different blocks. However, besides the benefit of serverless fog-cloud computing to run IoMT applications, many challenges are being further investigated. The tradeoff between cheap cost-and-demand QoS is a conflicting problem during execution. Due to the external services, the security of the offloaded data of different users has posed a challenge. Therefore, secure and cost-efficient task scheduling in an edge computing, serverless, decentralized system is becoming a challenge [12].

In this paper, the study investigates the cost-efficient task scheduling problem and security mechanism in the blockchain-enabled fog-cloud network [13]. The objective is to minimize the application cost during offloading and scheduling in the blockchain-enabled fog-cloud network. The study considers the workflow healthcare application, where the precedence-constraints sequence constrains all tasks. In the workflow, each task has a deadline and needs to be completed before a given threshold. The study considers the serverless, decentralized fog-cloud network, which consists of different functions which run inside containers. In order to solve the task scheduling problem in the IoMT workflow application, the study proposed the Blockchain-Enable Smart-Contract Cost-Efficient Scheduling Algorithm Framework (BECSAF), which consists of the following schemes: Smart-Contract-Scheme, Function Verification Pool, Task and Function Sequencing, Resource Matching, Smart-Contract Aware Blockchain-Enable Task-Scheduling. Algorithm 1 uses the BECSAF to solve the problem in different steps.
**Algorithm 1:** BECSAF.
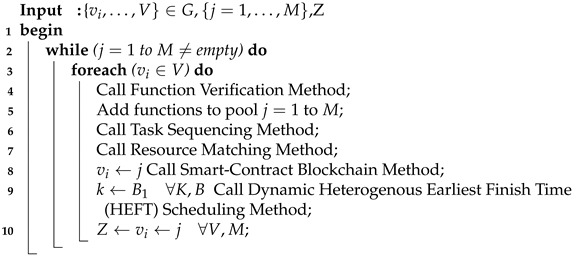


The study presents the following main contributions to the state of the art:

- Initially, the study devises the Blockchain-Enable Smart-Contract Cost-Efficient Scheduling Algorithm (BECSAF), consisting of the following schemes: Smart-Contract-Scheme, Function Verification Pool, Task and Function Sequencing, Resource Matching and Blockchain-Consensus-Scheme-Task-Scheduling. Algorithm 1 uses the BECSAF to solve the problem in different steps. The smart-contract scheme designates each connected node in IoMT to avoid any tampering with data in the network. The functions are the resource in IoMT; each function has different execution costs. Therefore, the study verifies the standard of each function before adding to them in the function pool;
- The study considers the applications that have stringent requirements for their execution, as well as the deadline and resources required for them to complete their process. Therefore, with different deadlines, the study implements task sequencing rules before scheduling them with nodes. The aim is to sort all requested tasks into topological order, and then to execute them in the optimized order;
- To adopt dynamic changes in the environment, the dynamic preemptive scheduler was suggested. The goal is to schedule tasks for the decentralized functions, minimize the execution cost, and meet the application deadline during processing in the system;
- To ensure the blockchain validity of distributed data and management of the load balancing situation, the resource leakage efficient blockchain-enabled schemes are devised to avoid any resource and disk-bound application failure in the system.

The remainder of this paper is organized as follows. Section 2 describes the problem description and problem formulation. Section 3 proposes the algorithm framework. Section 4 presents the simulation results in order to evaluate the performance of our algorithm. Section 5 concludes the summary and the intended future work.

## 2. Related Work

The Internet of Medical Things (IoMT) is a complex system that consists of different technologies (e.g., sensors, communication channels, and computing nodes at local and remote layers) to support healthcare mechanisms for patients. Three types of solution (e.g., static, dynamic and adaptive optimization) have attempted to solve the problem with offloading and scheduling techniques. Different studies achieved different objectives, as shown in Table 1. The problem parameter defines the considered problem through offloading, resource allocation and scheduling, along with the constraints and proposed methodology, according to the considered problem. Generally, all studies considered the fog-cloud network to easily manage the load-balancing between resources with different objectives. The gap-analysis highlights the aspects which studies did not consider in their IoMT application methodologies.

Baresi et al. [1] suggested a serverless-based IoMT system to minimize the resource cost of applications. The serverless system has a lower fog-cloud cost compared to virtual machines. However, they did not consider the security mechanism in the study. Eivy et al. [3], Adzic et al. [7], Adzic et al. [7] and Lynn et al. [8] and van et al. [9] suggested serverless based fog-cloud where instead of virtual machines they run functions inside containers. The objective is to minimize the execution and offloading cost of applications. These studies replaced the existing resource function provisioning methods and achieved multiple objectives, such as energy, delay, lateness and cost. These studies proposed the methodology based on static optimization (e.g., static application partitioning, static scheduling, static resource allocation) to solve the offloading, resource allocation and scheduling problem in the IoMT network. Rez et al. [10] suggested the serverless-based IoMT system could minimize the resource cost of applications. The serverless system has a lower fog-cloud cost compared to virtual machines. However, they did not consider the security mechanism in the study. Yan et al. [11], De-Lara et al. [12], Lakhan et al. [13] and Li et al. [14] and Li et al. [15,16] suggested serverless and container-based application partitioning, resource allocation and scheduling methodology-based linear and dynamic optimization in fog-cloud. The objective is to minimize the execution, energy, response time, and delay and offloading cost of applications. However, as mentioned earlier, these solved the scheduling and offloading problem without considering the security mechanism in the IoMT fog-cloud network.

The blockchain-enabled solution proposed in IoMT could save patient records in their original form. The primary goal of blockchain is to save data from tampering and offer immutable blocks in the distributed system. Many blockchain-enabled fog-cloud systems of IoMT have been suggested to guard the privacy and authenticity of patient data in the system. Lakhan et al. [17] suggested a blockchain-enabled system fora vehicular healthcare ambulance vehicle in the fog-cloud network. The miners in the consensus use symmetric encryption and decryption methods to save the data from network tampering. However, this work handles the fault-tolerant nodes within the execution runtime. The study’s objective is to minimize the security risk and cost of applications during offloading and scheduling in the system. Tariq et al. [17], Tariq et al. [18] and Islam et al. [19] devised a blockchain-enabled IoMT network using an Ethereum decentralized consensus to protect the patients’ IoT big data from tempering. These studies proposed methodologies based on dynamic optimization (i.e., dynamic scheduling) and adaptive optimization (e.g., reinforcement learning, both supervised and unsupervised)to solve the offloading and scheduling problem. The goal is to minimize the risk of data tampering in the distributed network.

The data-offloading-aware data allocation in the blockchain-enabled fog-cloud network system for healthcare sensors was proposed in [4,6,20,21,22,23,24]. These studies suggested a method to protect sensor data and enhances the performance of big-data analysis in the system. Dynamic and adaptive optimization heuristics, such as genetic algorithm and reinforcement aware schemes, were suggested [2,5,23,25,26,27]. The different objectives were obtained, such as the cost, security, response time and energy of sensors devices during offloading and scheduling in the IoMT network. All the studies used resource-provisioning methods and blockchain technology to achieve user cost, communication, and network security [28,29,30,31]. Table 2 represents the mathematical symbol for problem formulation.

## 3. Problem Description

**Figure 1:**
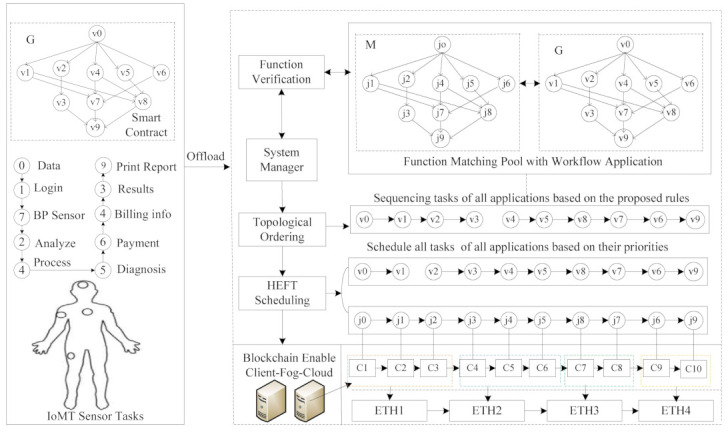
Smart-Contract Ethereum Aware Client-Fog-Cloud Assisted Healthcare System.

The study devises the cost-efficient scheduling IoMT system based on the blockchain-enabled fog-cloud network, as shown in Figure 1. The proposed consists of different components: application-level smart-contract, Function Verification, System Manager, Task Sequencing, Task Scheduling, Blockchain-Enable fog-cloud network. The fog cloud offers published functions of the different cloud providers at various specifications. The providers are IBM OpenWhisk, AWS Lambda, Azure Functions, Google Cloud Functions, Alibaba Function Compute, and Kubeless Functions that offer functions, and everything manages by themselves. The System Manager controls all components in the system.

Function, as a service, is a serverless edge computing services category that offers a forum for customers to create, operate, and manage application functionalities without the difficulty of building and maintaining the infrastructure that is usually associated with the creation and launch of an application.

### 3.1. System Model

**Figure 2:**
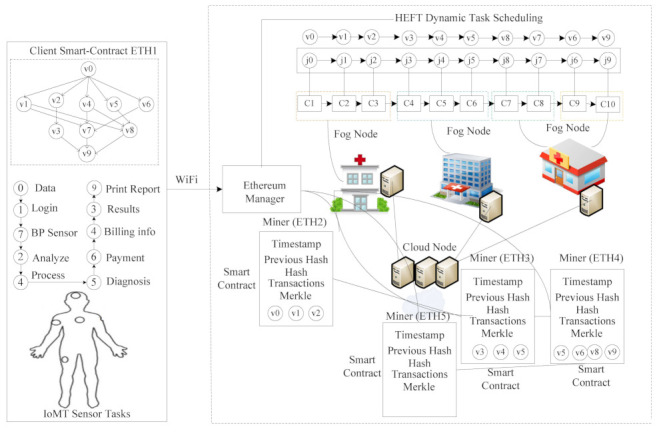
System Model.

The proposed framework consists of the application layer and resource layer, as shown in Figure 2. The application layer is composed of workflow tasks and body sensors. However, the application layer offloads all workflow tasks to the fog-cloud system for further execution due to resource-constraint sensors. Due to security issues at the communication layer, the smart contract is implemented at the application layer, predicting the network situation. If the data size and duration of offloading are greater than their given time and size, the smart contract will generate security to the application layer. Otherwise, if the situation appears to be normal, the whole application will offload to the fog-cloud system.

The resource layer combines the distributed fog nodes and remote cloud, as shown in Figure 2. All the fog nodes communicate with each other via different communication channels. All hospital fog nodes are directly connected to the remote cloud. The System Manager is the primary controller in the system, and handles all execution process inside the system. Blockchain Management creates blocks (e.g., miners) for all transactions at each fog node with different elements. For instance, Ethereum miner (ETH1) is configured with smart-contract, Timestamp, Previous Hash, Hash and Transaction Merkle of $v_{0},v_{1},v_{2}$ transactions. Conversely, miner (ETH2), miner (ETH3), miner (ETH4) and miner (ETH5) use the same elements to achieve a secure transaction between client–fog–cloud nodes. The fog cloud executes workflow tasks $\left\{v_{0},\ldots ,v_{9}\right\}$ based on the matched functions $\left\{j_{0},\ldots ,j_{9}\right\}$ from the pool based on the proposed scheduling scheme. The user application assumes that the thin-client and fog-cloud nodes are thick-client. Therefore, a smart contract is a level of agreement between thin-client and thick-clients during offloading and scheduling in the system.

### 3.2. Problem Formulation

The IoMT workflow application is represented by the directed acyclic graph, i.e., G(V,E). For the two tasks $v_{i},v_{z}\in V$, an edge $e(v_{i},v_{z})\in E$ represents the data dependency between task $v_{i}$ and task $v_{z}$, which means that $v_{i}$ should complete its execution before $v_{z}$ starts. The application *G* has *N* number of tasks. Task $v_{0}$ is the entry task and $v_{n}$ is the exit task. We use $data_{i}$ to denote the original data volume of task $v_{i}$, whereas, $data_{i,z}$ denotes the generated data volume from task $v_{i}$ to $v_{z}$. Each task $v_{d}i$ has a deadline inside the workflow during its processing in the system.

The fog-cloud nodes are represented by {k=1,…,K. Each computing node can create the number of containers, i.e., $\{C_{1},\ldots ,C$}. Each node configured with the Ethereum blockchain consensus blocks, i.e., $\left\{ETH_{1},\ldots ,ETH\right\}$. The functions pool for the tasks of different cloud vendors is represented by $M_{i}=\left\{M_{i}^{0},M_{i}^{1},\ldots ,M_{i}^{|M_{i}|-1}\right\}$. $M_{i}^{jCk}$ is the $j^{th}$ function of node *k* for $v_{i}$, which is executed inside the container. $B_{ijCk}$ is the start time of a task at the $j^{th}$ function in the $k^{th}$ node, and $F_{ijCk}$ is the finish time of the $S_{ijk}$. The execution time of a task is calculated by $T_{ijCk}^{e}$. The cost of each task is determined in the following way: $Cost_{ijCk}$ is illustrated by the $S_{ij}=\left\{T_{ij}^{e},Cost_{ijCk}\right\}$. The binary assignment of each task $v_{i}$ to the available function is determined as follows.

(1) $$x_{ijCk}=\left\{\begin{array}{cc} 1, & S_{ijCk}\;\mathrm{function}\;\mathrm{chooses}\;\mathrm{for}\;v_{i}\\ 0, & \mathrm{otherwise}, \end{array}\right.$$

Equation (1) determines the binary assignment of tasks to the functions.

(2) $${\mathrm{Smart}}_{data_{i,z}}=\left\{\begin{array}{cc} 1, & \sum_{e=1}^{E}Smart_{data_{i,z}}\;\mathrm{if}\;\mathrm{tasks}\;\mathrm{data}-\mathrm{size}\;\mathrm{equal}\\ 0, & \mathrm{Tempered}, \end{array}\right.$$

$Smart_{data_{i,z}}$ determines the smart-contract rules during communication between tasks and offloading, as determined in Equation (2), whereas, $\sum_{e=1}^{E}data_{i,z}$ is the communication of tasks between thin-client and thick-client. The objective is to reduce cost of workflow tasks under their deadline constraints. The considered problem is formulated as follows.

(3) $$\min Z=\sum_{v_{i}=1}^{V}\sum_{j=1}^{|M_{i}|}\sum_{k=1}^{K}\sum_{C=1}^{C}Cost_{ijCk}\times x_{ijCk}.$$

*Z* represents the objective function of the study, as defined in Equation (3). Subject to

(4) $$\sum_{j=1}^{|M_{i}|}\sum_{k=1}^{K}x_{ijCk}=1,\;\forall v_{i}\in V.$$

Each task is assigned to only function at a computing node, as defined in Equation (4).

(5) $$F_{ijCk}=\sum_{j=1}^{|M_{i}|}\sum_{k=1}^{K}B_{ijCk}+T_{ijCk}^{e}\times x_{ijCk}\leq d_{i},\;\forall v_{i}\in V,$$

The finish time of tasks must be within their deadlines, defined in Equation (5).

## 4. Proposed Schemes

The study proposed the Smart-Contract Aware Blockchain-Enable Cost Scheduling Algorithm (BESCAF), consisting of the following schemes: Smart-Contract-Scheme, Function Verification Pool, Task and Function Sequencing, Resource Matching, Blockchain-Consensus-Scheme-Task-Scheduling. Algorithm 1 uses the BECSAF to solve the problem using the following steps.

### 4.1. Function Verification

This study verifies each vendor function based on different standards and rules, as described in Table 3. All functions share their data through the Simple Object Access Protocol due to the previous limitations of UAV workflow applications (SOAP). The data communication format should be in the form of a JavaScript Object Notation (JSON). In terms of fault operation, the provider can comply and offer an alternative service (e.g., available mood). The time complexity, i.e., O(n×n) with various memories, should be optimal (e.g., 512–1024–2048 MB). The execution cost must be measured in milliseconds for each feature.

### 4.2. Topological Ordering of Tasks

The system initially sorted all tasks based on their deadlines. We sorted all tasks based their deadlines and cost. Algorithm 2 shows the pseudo-code of the ranking of the requested tasks into some topological order.
**Algorithm 2:** Topological Ordering Scheme.
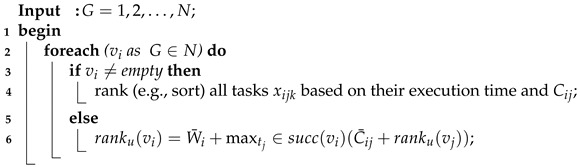


### 4.3. Resource Matching

The paper introduces the resource-matching method which determines the function of different tasks before scheduling the system to sort cost-efficient functions in descending order, at 10 min intervals, from the service pool. The IoMT system can add thousands of functions to the services pool; the algorithm sorts all the best services in terms of cost, in descending order, due to their fast matching time. Algorithm 3 uses the task preference and function preferences as inputs. Based on the cost and task requirements, the algorithm creates the match list, where each task is assigned to a function that can satisfy its needs. In the end, it matches all tasks until the list of tasks becomes empty.
**Algorithm 3:** Resource Matching.
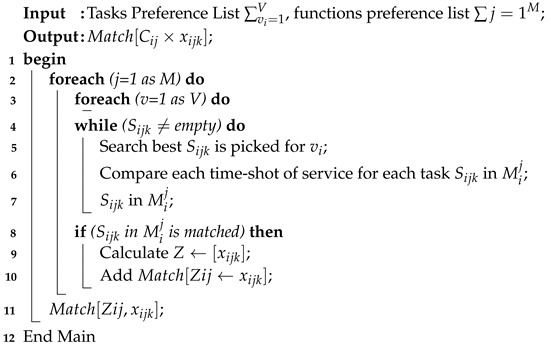


### 4.4. Smart-Contract Aware Ethereum and HEFT Dynamic Scheduling

The smart-contract-enabled client–fog–cloud blockchain performs secure data transactions to different nodes, with the same rules and regulation. In the study, the smart-contract ethereum blockchain performs secure transactions in different steps, as shown in Algorithm 4.
**Algorithm 4:** Ethereum Smart-Contract-Blockchain.
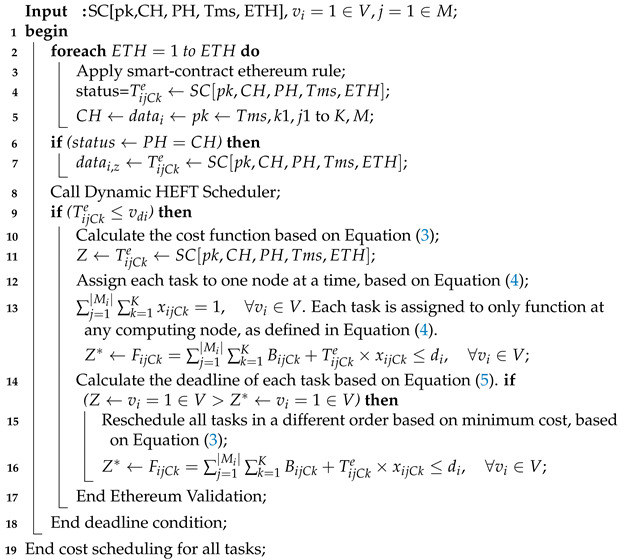


**Figure 3:**
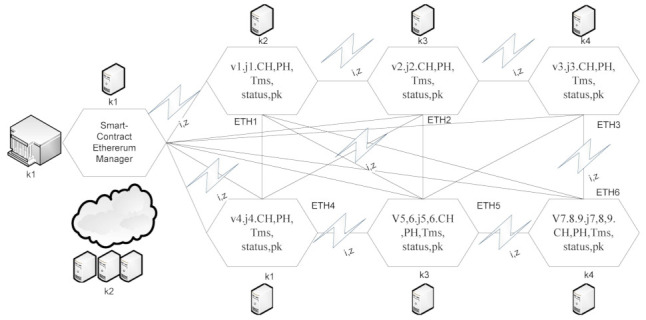
Smart-Contract Ethereum Mechanism in Distributed Client-Fog-Cloud.

Algorithm 4 can be defined in the following steps:

- According to the mechanism defined in Figure 3, each task required data, i.e., $data_{i,z}$ for its execution on different computing nodes, e.g., k=1∈K. Each node performs a transaction for each task based on the smart-contract rule that the data should be encrypted in the current ethereum using a public key, which the ethereum manager issues. The data sharing between task $v_{i}$ and task $v_{z}$ must be validated by the proof-of-work method, as defined in steps 6 to 8;
- Each ethereum can perform hashing based on a public key with 128 bits, with the share encrypted data from node k1 to node k2. Node $k_{2}$ initially decrypts the previous hash PH into plain-text, and this is executed on node k2. After that, the task $v_{3}$ scheduled at node $k_{3}$ is that the previously matched node should be matched in the node $k_{3}$ of previously executed data during precedence constraint data-sharing between tasks $v_{vi,vz}$;
- Figure 3 shows that each ethereum transaction performs and save the data at the particular node, using a function inside the container;
- The function offers CPU resources, data-storage and memory to run any transaction of the task before offloading to another node for further processing;
- If the current hash CH of any data which are offloaded to another node must be matched with the PH in the particular timestamp Tms;
- Each ethereum ETH can execute multiple transactions, as shown in Figure 3;
- Algorithm 4 perform a secure ethereum transaction based on the public between different computing nodes, without any loss of generosity.

The study scheduled all tasks based on the dynamic Heterogeneous Earliest Finish Time (HEFT) rules, as defined in Equation (5). After meeting the deadline, the scheduler reschedules all tasks based on the optimal execution cost $Z^{*}$, based on Equation (3).

## 5. Performance Evaluation

The simulation parameters were implemented in the serverless evaluation model defined in Table 4.

Table 5 consists of these primitives: Providers, Task, Function, Node, Cost (Memory (MB), ${}^{*}$Execution (ms)). The vendors offer different functions to execute tasks, known as function as a service. However, these functions must run inside containers at other computing nodes, such as fog and cloud computing. Therefore, the study considered fog-cloud nodes to execute functions based on user requirements in the IoMT.

Table 5 shows the cost of functions of different vendors. Each of the functions was deployed using the Python 3 runtime with 256 MB of memory. The first generated benchmark function was a factorial function, which calculates the resulting returning factorial 150 times.

### 5.1. System Implementation

**Figure 4:**
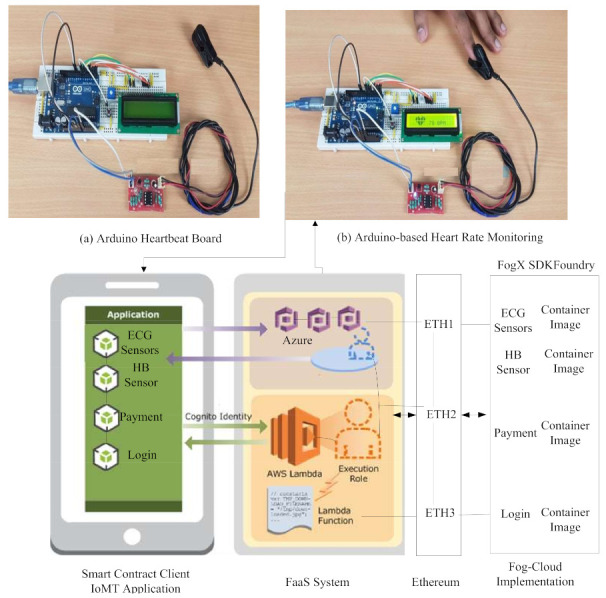
Smart-Contract-Ethereum Enable Client-Fog-Cloud Assisted System for IOMT.

The function as a service-based serverless system implemented the components shown in Figure 4.

The system consists of application layer which offers an interface for the user to initialize their tasks. The resource layer provides functions that are implementied inside containers with an edgex-foundry mechanism. The blockchain is implemented inside the system, as shown in Figure 4, to maintain applications in the distributed environment. We add the functions of two vendors, such as Amazon and Azure, to the systems. FaaS are the functions defined in Table 5. The goal is to execute all tasks on functions with the blockchain-enabled network. The smart-contract and ethereum blockchain ETH are implemented, as shown in Figure 4. The goal is to execute all transactions within blocks of ethereum without any tempering, as shown in Figure 3.

#### 5.1.1. IoMT Sensors

The Heartbeat Sensor is an electronic system used to measure heart rate, i.e., heartbeat velocity. Body temperature control, heart rate and blood pressure are the basic things we do to keep ourselves safe. We use thermometers and a sphygmomanometer to monitor arterial pressure or blood pressure, to calculate body temperature. It is possible to track the heart rate in two ways. The first is to manually check the pulse of the wrists or neck. The second is to use a Heartbeat Sensor. In this project, we have developed a Heart Rate Monitor Device using Arduino and Heartbeat Sensor. The Heartbeat Sensor Concept, the Heartbeat Sensor and the Arduino-Based Heart Rate Monitoring Device, identified using a functional heartbeat sensor. For athletes and patients, controlling heart rate is very important as it determines the state of the heart (just heart rate). There are many methods of calculating heart rate, and electrocardiography is the most reliable. However, using the Pulse Sensor is the best way to track the heart rate. It comes in various shapes and sizes, and offers a quick way to calculate the pulse. Wrist Watches (Smart Watches), Smart Phones, chest belts, etc., are available with heartbeat sensors. The heartbeat is measured in beats per minute or bpm, representing the number of times in a minute that the heart contracts or expands.

#### 5.1.2. IoMT Application

We designed the android IoMT application, which consists of four types of sub-applications, such as cancer-aware monitoring, Heartbeat, ECG, and EEG monitoring. These applications consist of workflow tasks, as shown in Figure 4, and different functions are required to run them. All sensors are connected with an android mobile phone. The mobile phone was connected to the proposed system, which offers services based on different vendor functions and processes them inside containers. The EdgeX Foundry is exploited to design the basic infrastructure of the applications.

#### 5.1.3. Edgex Foundry

EdgeX Foundry is a Linux Foundation-hosted, a vendor-neutral open-source platform offering a popular mobile framework for IoMT edge computing. There is a series of loosely connected functions of different vendors, grouped into different layers inside containers.

### 5.2. Baseline-Approaches

For analysis of the results, the performances of existing systems and the proposed system were evaluated based on resource and application execution in terms of cost and deadline (QoS). The study implemented three systems with a similar architecture, but, somehow, the resources are different, as is their usage in IoMT workflow applications. The baseline approaches are discussed in the following way.

- Baseline1: Baseline2: The existing studies [16,17,18,19,20,21,22,23,24] suggested a blockchain-enabled fog-cloud network for healthcare applications. These works considered the containers and virtual machines at any computing node as the resource. The cost model based on different resource-provisioning (on-demand, on-reserve, spot-instance) was implemented to execute the applications. However, the study only considered the on-demand resource-provisioning for the application execution in the performance evaluation part. There are many components to the existing proposed systems, for instance, offloading, resource allocation, blockchain-enabled chaining and smart-contract rules. Therefore, the performance evaluation shows the schemes’ performance in terms of cost for healthcare application via different systems.

### 5.3. Result Discussion

The execution cost of each application depends upon the usage functions and their properties. For instance, each function has a different memory and execution time. Therefore, the execution cost of each function is additional during its execution. The study executed all tasks within their deadlines with a lower cost than existing techniques. The proposed serverless, decentralized-based fog-cloud could run healthcare applications, fulfilling their quality-of-service requirements. It is hard to balance execution cost and deadlines during scheduling and offloading in the distributed blockchain-enabled fog-cloud network. There are many risk factors in distributed computing, such as failure, security attack, missing deadlines, execution cost and total execution time. Therefore, the study evaluated the performances of existing systems based on the following metrics: failure, security attack, deadline missing, execution cost and total execution time.

**Figure 5:**
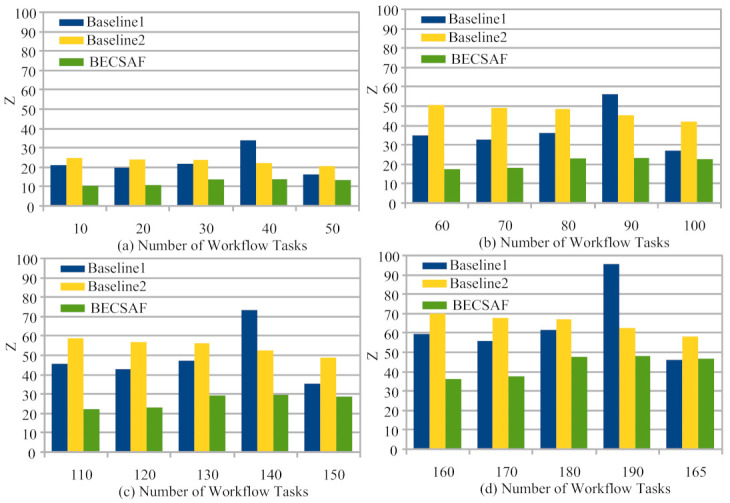
IoMT Workflow Application Execution Cost in Fog-Cloud System.

The execution cost of each application depends upon the usage functions and their properties. For instance, each function has a different memory and execution time. Therefore, the execution cost of each function is increased during its execution. The study executed all tasks within their deadlines with minimal cost compared to existing techniques. The proposed system meets the quality-of-service requirements of the application. It is hard to balance execution cost and deadlines during scheduling and offloading in the distributed blockchain-enabled fog-cloud network. There are many risk factors in distributed computing such as failure, security attack, missing deadlines, execution cost and total execution time. Therefore, the study evaluated the performances of existing systems based on the following metrics: failure, security attack, deadline missing, execution cost and total execution time. In the fog-cloud network, the tasks’ deadlines also have a critical role in the system. For instance, the healthcare monitoring system uses different, life-critical sensors (e.g., heartbeat, blood-pressure, location-monitoring of an ambulance). Therefore, each task has a critical deadline for its completion or a response from the fog-cloud system. In the second metric, the performance evaluation is analyzed based on the deadline (QoS) of application tasks. Therefore, the task deadlines during scheduling are essential, as well as the cost. If the system responds late, the critical patients who use the heartbeat sensor during critical rating can suffer from any health issue. Figure 5a–d shows that the BECSAF has fewer missed non-critical fewer tasks during processing in the system when considering critical healthcare issues. Existing baseline approaches (Baseline1 and Baseline2) only considered the offloading performance and ignored the system performance in terms of the deadline, leading to many missed critical task deadlines. Therefore, BECSAF considered this important prospect during the processing of application tasks in the system. Figure 5a shows the performances of schemes with the 150 workflow tasks. The proposed method outperforms all existing techniques for workflows with 50, 100, 150 and 165 tasks. The main reason for this is that all existing schemes only considered the resource-allocation strategies with latency requirements. However, existing works did not focus on workload deadline, priority and execution in the IoMT network. Without task sequences, function validation, and dynamic scheduling, IoMT tasks can suffer from lower performances in IoMT.

### 5.4. Fault-Tolerant

The failure-aware system always reduces the execution cost of applications in the IoMT network. In comparison, there are many types of failure in the system. For instance, the system’s failure consists of transient failure, application failure, network failure, and node failure. However, existing studies did not consider the blockchain failure situation when blocks are overloaded, or data-tempering occurs in IoMT. The study finds the failure-recovery cost in IoMT, which is included in system’s execution cost. Therefore, it should be taken by the system as the cost-constraint for workflows. The fault-tolerant aware blockchain-enable fog-cloud network is preliminary in the serverless, decentralized system. There are many failure possibilities in each node, such as failure of the computing node due to over-balancing, and many reasons for these. Therefore, a failure-aware system can handle any failure transaction in the blockchain-enabled fog-cloud network. This work proposed a Practical Aware-Byzantine-Fault-Tolerance scheme that operates any failure in the system. The loss has a lot of impact on the application cost during scheduling, and if failure remains untreatable, it will lead to an increased recovery cost for the application in the system. Therefore, the study implemented the Practical Aware-Byzantine-Fault-Tolerance scheme in the blockchain–fog–cloud and analyzed the failure ratio of tasks in the system. The study examined the failure-aware performance of the applications and design in different layers and explained the following cases.

Cost of Failure Between User Application *G* to Request Computing Node *K*.

Cost of Failure Between Fog Node $k_{1}$ to $k_{2}$ During Data Travelling.

Cost of Failure Between Fog Node $k_{2}$ to $k_{3}$ During Data Travelling.

The cost of Failure Between Fog Node $k_{3}$ to $k_{4}$ During Data Travelling. The detail of the failure defined in the following.

**Figure 6:**
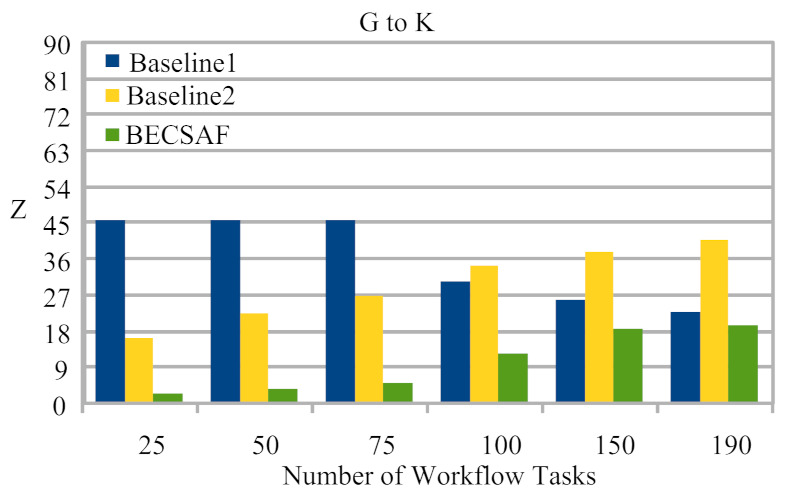
Cost of Failure Between User Application *G* to Request Computing Node *K*.

**Figure 7:**
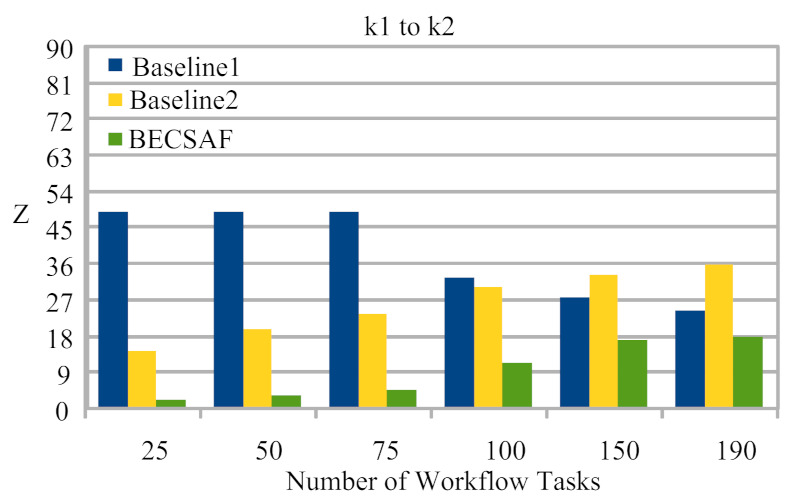
Cost of Failure Between Fog Node $k_{1}$ to $k_{2}$ During Data Travelling.

**Figure 8:**
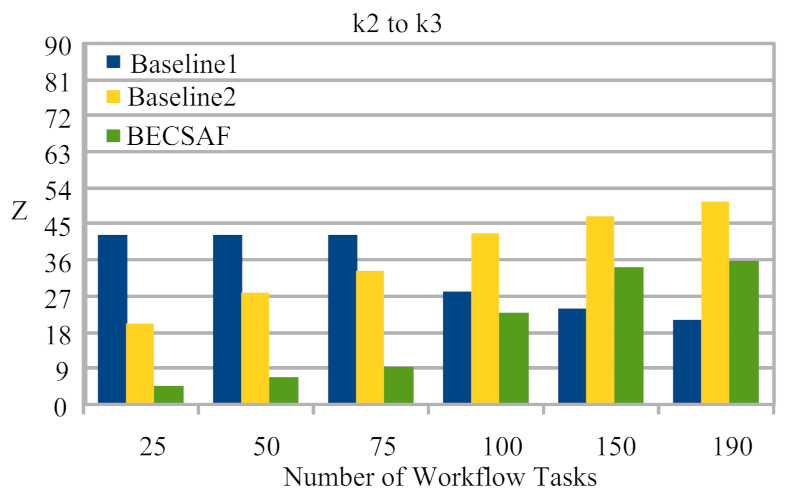
Cost of Failure Between Fog Node $k_{2}$ to $k_{3}$ During Data Travelling.

**Figure 9:**
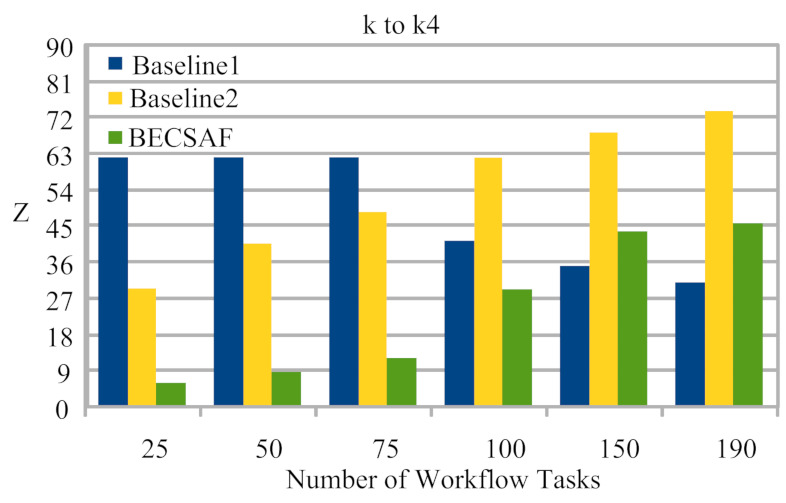
Cost of Failure Between Fog Node $k_{3}$ to $k_{4}$ During Data Travelling.

- Case-1: The failure between application *G* and requested node *k*. The users can request any computing node in the fog-cloud network for processing. The request failure or process failure to be analyzed are based on a security attack, calculated based on data size. If the generated tasks’ data, or original data size, increases in terms of its actual size, then the transaction fails. If the communication or computing fails, then the failure cost is incurred during the recovery process. The proposed BECSAF system detects the failure in advance, before offloading the system based on the smart-contract scheme, which identifies any failure before sending it to any node. Therefore, Figure 6 shows that BECSAF incurred the lowest failure cost between the user application and initial computing node during the process;
- Case-2: The failure between computing node $k_{3}$ and computing node $k_{4}$ during data travelling for further execution. The request failure or process failure is analyzed based on the security attack, calculated based on data size. If the generated data of tasks, or original data size, increases in terms of its actual size, then the transaction fails. The proposed BECSAF system detects the failure in advance before offloading the system based on the smart-contract scheme, which identifies any loss before sending it to any node. Therefore, Figure 7 shows that BECSAF incurred the lowest failure cost between the user application and initial computing node during the process;
- Case-3: The failure between computing node $k_{2}$ and computing node $k_{3}$ during data travelling for further execution. The request failure or process failure is analyzed based on a security attack, calculated based on data size. If the generated data of tasks or original data size increase in terms of actual size, then the transaction fails. The proposed BECSAF system detects the failure before offloading the system based on the smart-contract scheme, which identifies any failure before sending at any node. Therefore, Figure 8 shows that BECSAF incurred the lowest failure cost between the user application and initial computing node during the process;
- Case-4: The failure between computing node $k_{3}$ and computing node $k_{4}$ during data travelling for further execution. The users can request any computing node randomly in the fog-cloud network for processing. If the generated data of tasks or original data size increase in terms of its actual size, then the transaction fails. The proposed BECSAF system detects the failure before offloading the system based on the smart-contract scheme, which identifies any failure before sending it to any node. Therefore, Figure 9 shows that BECSAF incurred the lowest failure cost between the user application and initial computing node during the process.

### 5.5. Blockchain-Enable Fog-Cloud Performance

**Figure 10:**
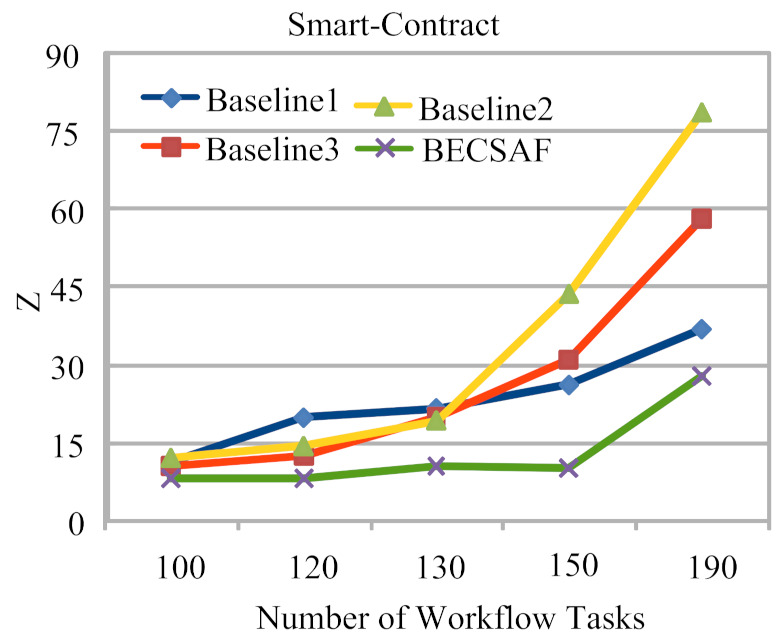
Smart-Contract.

In the distributed computing, the study organized the cost and deadline performances of the proposed blockchain-enabled fog-cloud system into different levels: user-level, node-to-node level and fog-to-cloud level. Initially, the application offloads the entire application workload to the fog-cloud system in a secure way. The smart-contract scheme detects the execution size before sending it to the fog-cloud system in advance. If the offloaded application tasks have different data sizes, the smart-contract method generated the failure or attack message to the system and application. In this way, advanced failure detection can minimize the failure or attack cost of healthcare applications. Figure 10 shows that the BECSAF outperforms all baseline approaches in terms of attack or failure for IoMT workflow application in the network.

During the sharing of data in the fog-cloud network, the following attributes should be present: authenticate, secure, double spending, and data validation. Therefore, this study implemented the blockchain Blockchain-Consensus Scheme to handle all attributes in the system.

**Figure 11:**
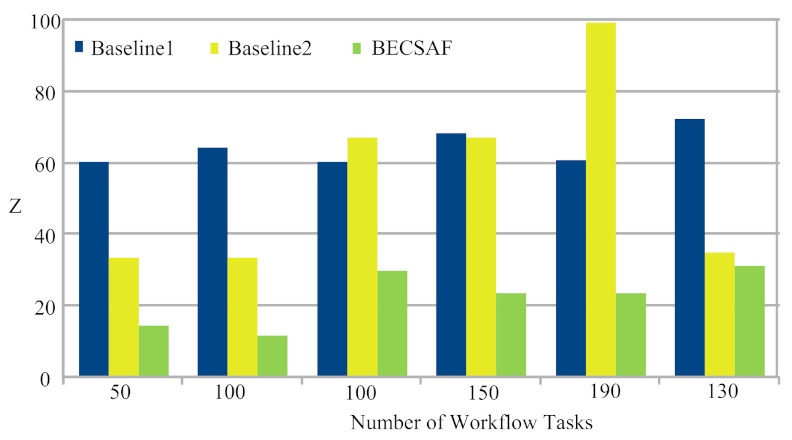
Proof of Sake for IoMT workflow Transactions in Blockchain-Enabled Fog-Cloud Network.

**Figure 12:**
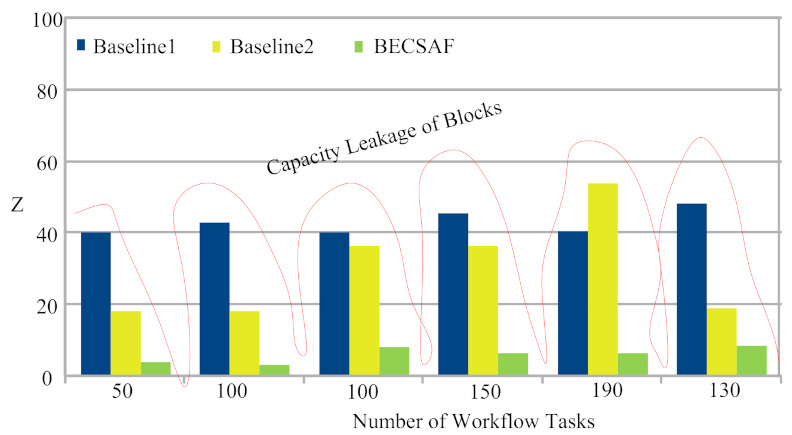
Resource Capacity Leakage of Blocks in Blockchain.

- Smart-Contract The study implemented smart-contract rules for all blockchain-miners, which can execute many transactions inside the same block during processing. The smart-contract rules avoid any violation, such as double-spending, transaction and data tampering.
- Sharing Data: All blocks share each transaction data and verify the hashing of the previous block before performing the new transaction for the requested tasks.
- Block-Resource Leakage. Each block has limited resource space, therefore, there could be leakage if the requested transactions increase their limit. This overloading or leakage will lead to longer matching or authenticate cost for all blocks in the blockchain network. Figure 11 and Figure 12 shows the BECSAF of all transactions of the same application in different blocks controlled based on the proposed scheme and incurred with lower blockchain cost.

Therefore, controlling resource leakage in the blockchain is a necessary job during blockchain-processing for different applications in the system.

## 6. Conclusions and Future Work

The study devised the novel, cost-efficient and secure IoMT system based on the blockchain-enabled fog cloud. The study’s goal is to minimize the cost of the healthcare application services during processing in the system. The performance evaluation results show that the suggested IoMT system outperforms the existing baseline healthcare systems in terms of cost and security in the distributed healthcare system. Many metrics were evaluated, such as execution cost, deadline, security, fault-tolerance, and resource-leakage during evaluation. From the analysis of the results, the proposed study improved the cost of the of the IoMT application services and provided a secure distributed environment for execution.

In future work, the study will focus on mobility-aware IoMT services with familiar dynamic learning approaches for the different healthcare applications: drone-ambulance system and Internet of Unmanned Healthcare Vehicle Things network. Knowledgeable mobility services are always valuable for the self-adaptive and dynamic environment of healthcare applications.

## Figures and Tables

**Table 1:** Existing Blockchain-Enable IoMT System and Objective.

Study	Problem	Constraints	Methodology	Objective
[1]	Offloading	Energy	Static Optimization	Min.Power
[3]	Offloading	Delay	Static Optimization	Min.Delay
[7]	Offloading	Tardiness	Static Optimization	Min.Delay
[8]	Allocation	Tardiness	Static Optimization	Min.Tardiness
[9]	Resource	Lateness	Static Optimization	Min.Tardiness
[10,11]	Scheduling	Execution-Time, cost	Linear-Optimization	Lateness
[12,13,14]	Scheduling	Execution-Time, cost	Dynamic-Optimization	Scale-up
[15]	Offloading	Security	Dynamic-Optimization	Min.Risk
[16]	Task Alloc.	Security	Dynamic-Optimization	Min.Risk
[17]	Task Alloc.	Security	Dynamic-Optimization	Min.Risk
[18]	Resource Alloc.	Security	Dynamic-Optimization	Min.Risk
[19]	Offloading	Security	Dynamic-Optimization	Min.Risk
[20]	Task Alloc.	Security	Adaptive-Optimization	Min.Cost
[21,22]	Task Alloc.	Security	Adaptive-Optimization	Min.Cost
[23]	Offloading	Security, cost	Adaptive-Optimization	Min.Risk
[24]	Offloading	Security	Dynamic-Optimization	Min.Risk
Proposed	Scheduling	Security, cost	Dynamic-Optimization	Functions

**Table 2:** Mathematical Notation.

Notation	Description
*G*	IoMT workflow application
*V*	Number of application tasks *G*
$v_{i}$	$i^{th}$ workflow application task *G*
$v_{id}$	The task deadline $v_{i}$
*K*	Number of fog-cloud computing nodes
*k*	The $k^{th}$ computing node of *K*
$\epsilon_{k}$	The resource capability of $k^{th}$ node
*M*	Pool of functions
*j*	$j^{th}$ function of node *k*
*C*	Total number of containers in node *k*
$C_{k}$	The $C^{th}$ container of node *k*
*B*	Number of blocks in the blockchain
$B_{1}$	The $i^{th}$ block of *B*
$B_{capacity}$	Capacity of block *B*

**Table 3:** Rules and Standard for Functions to be Part of Proposed System.

Services Functions	Standards	RunTime	Vendor	Failure	Complexity	Memory	Execution
$j_{1}$	SOAP	JSON	Azure	Availability	O(n×n)	512–1024 MB	Milliseconds
$j_{2}$	SOAP	XML	Amazon	Availability	O(n×n)	512–1024 MB	Milliseconds
$j_{3}$	SOAP	XML	AliBaba	Availability	O(n×n)	512–1024 MB	Milliseconds
$j_{4}$	SOAP	JSON	IBM	Availability	O(n×n)	512–1024 MB	Milliseconds
$j_{5}$	SOAP	JSON	Kubless	Availability	O(n×n)	512–1024 MB	Milliseconds
$j_{6}$	SOAP	JSON	Google	Availability	O(n×n)	512–1024 MB	Milliseconds
$j_{7}$	SOAP	XML/JSON	Azure	Availability	O(n×n)	512–1024 MB	Milliseconds
$j_{8}$	SOAP	JSON	Amazon	Availability	O(n×n)	512–1024 MB	Milliseconds
$j_{9}$	SOAP	JSON	Azure	Availability	O(n×n)	512–1024 MB	Milliseconds

**Table 4:** Simulation Parameters.

Simulation Parameters	Values
Windows OS	Linux Amazon GenyMotion
Sensors	Heartbeat and Blood-Pressure
Centos 7 Runtime	X86-64-bit AMI
Languages	JAVA, XML, Python
Android Phone	Google Nexus 4, 5, and 7S
Experiment Repetition	160 times
Simulation Duration	12 h
Simulation Monitoring	Every 1 h

**Table 5:** Function of Different Vendors.

Providers	Task	Function	Node	Cost (Memory (MB) × Execution (ms))	*Z*
IBM OpenWhisk	$v_{1}$	$j_{1}$	$k_{1}$	512 (MB)	0.3
IBM OpenWhisk	$v_{2}$	$j_{2}$	$k_{1}$	1024 (MB)	0.7
IBM OpenWhisk	$v_{3}$	$j_{3}$	$k_{1}$	2048 (MB)	0.11
AWS Lambda	$v_{4}$	$j_{4}$	$k_{2}$	512 (MB)	0.5
AWS Lambda	$v_{1}$	$j_{5}$	$k_{2}$	1024–2048 (MB)	0.4–0.9
Azure Functions	$v_{6}$	$j_{6}$	$k_{3}$	1024 (MB)	0.8
Azure Functions	$v_{7}$	$j_{7}$	$k_{3}$	2048 (MB)	0.14
Google Cloud Functions	$v_{8}$	$j_{8}$	$k_{3}$	1536 (MB)	0.17
AliBaba Function Compute	$v_{9}$	$j_{9}$	$k_{3}$	2048 (MB)	0.16
Kubeless Functions	$v_{10}$	$j_{10}$	$k_{1}$	4096 (MB)	3

## Data Availability

All the experimental data are generated at the local institution servers. Therefore, it cannot be made publicly available for other researchers.
